# Prognostic Factors Influencing Postoperative Survival in Patients With Neuroendocrine Carcinoma of the Bladder: A Population‐Based Study

**DOI:** 10.1002/cam4.70758

**Published:** 2025-03-10

**Authors:** Liang Liu, Pan‐Ying Zhang, Yu Xiao, Qiang Wang, Ji Zheng

**Affiliations:** ^1^ Department of Urology, Key Laboratory of Molecular Pathology and Early Diagnosis of Tumor in Hebei Province, Prostate & Andrology Key Laboratory of Baoding Baoding No. 1 Central Hospital Baoding China; ^2^ Department of Urology Hebei General Hospital Shijiazhuang China; ^3^ Psychosomatic Medical Center, The Fourth People's Hospital of Chengdu Chengdu China; ^4^ Psychosomatic Medical Center, The Clinical Hospital of Chengdu Brain Science Institute, MOE Key Lab for Neuroinformation University of Electronic Science and Technology of China Chengdu China; ^5^ Department of Urology, Urologic Surgery Center Xinqiao Hospital, Third Military Medical University (Army Medical University) Chongqing China; ^6^ State Key Laboratory of Trauma and Chemical Poisoning Third Military Medical University (Army Medical University) Chongqing China

**Keywords:** neuroendocrine carcinoma, prognosis, propensity score matching, urinary bladder neoplasms, urothelial carcinoma

## Abstract

**Background:**

This study aimed to identify prognostic factors influencing survival in patients with bladder neuroendocrine carcinoma (NC).

**Methods:**

This study utilized the Surveillance, Epidemiology, and End Results (SEER) database (2004–2015) to compare NC with urothelial carcinoma (UC). We evaluated the prognostic value of clinicopathological characteristics and survival outcomes for bladder NC patients. Multivariable Cox proportional hazard models and propensity score matching (PSM) were employed for analysis.

**Result:**

A total of 99,704 patients were included, with 603 in the NC group and 99,101 in the UC group. Compared with the UC group, the NC group was inclined to receive radical cystectomy (34.2% vs. 12.2%), radiation (25.0% vs. 4.6%) and chemotherapy (62.0% vs. 22.2%) as treatment options. Multivariate Cox regression analysis revealed higher overall survival (OS) and cancer‐specific survival (CSS) outcomes for NC patients who underwent radical cystectomy (HR = 0.569, 95% CI = 0.537–0.603, *p* < 0.001; HR = 0.531, 95% CI = 0.489–0.577, *p* < 0.001; respectively). To mitigate bias, a 1:1 propensity score‐matched analysis was performed on both groups, resulting in 1202 patients (*n* = 601 per group). Multivariate Cox regression analysis identified seven risk factors for OS and CSS: age at diagnosis, race, cT stage, cN stage, cM stage, histological type, and chemotherapy. Additionally, surgery of the primary site was a prognostic factor for OS. A better prognosis was observed for NC patients who underwent radical cystectomy compared to those who did not. NC patients who only received radical cystectomy have a better prognosis in both OS (log‐rank *p* = 0.002) and CSS (log‐rank *p* = 0.009) compared with those who only received radiotherapy.

**Conclusion:**

Age, race, TNM stage, chemotherapy, and surgery were identified as independent predictors of bladder NC patients. Radical cystectomy may represent the optimal therapeutic approach to improve the prognosis of NC patients.

## 1 Background

Bladder cancer (BCa) was divided into non‐muscle‐invasive bladder cancer, which frequently recurs but slowly progresses, and muscle‐invasive bladder cancer, with the characteristics of rapid progress and a high degree of malignancy and mortality rates. It is the most common urologic malignancy in the United States, with an estimated 81,000 new cases diagnosed in 2022 [1]. BCa has a complex etiology with multiple contributing factors. However, despite this knowledge, few of these factors have been successfully leveraged to meaningfully reduce the prevalence of the disease. Treatment for BCa is primarily directed at urothelial carcinoma (UC), the most common pathological type. Among all bladder cancers, neuroendocrine carcinomas (NC) are relatively rare, accounting for less than 1% [2]. This category includes large‐cell neuroendocrine carcinomas, small‐cell neuroendocrine carcinomas, and mixed neuroendocrine‐non‐neuroendocrine neoplasia. Small‐cell neuroendocrine carcinoma is the most common type of NC and is associated with a poor prognosis. Compared to mixed forms or pure urothelial carcinomas, patients with pure small‐cell neuroendocrine carcinomas have significantly poorer outcomes, with a 5‐year survival rate ranging from 8% to 16% [3, 4]. Bladder NC is more common in men over 50 years old. It exhibits neuroendocrine differentiation and immunohistochemical characteristics that distinguish it from urothelial carcinoma. NC of the bladder often expresses neuron‐specific enolase (NSE), chromogranin A, and synaptophysin, which are markers of neuroendocrine differentiation [5]. Additionally, NC and UC can be differentiated using other immunohistochemistry markers. It is now understood that NC of the bladder typically expresses p16 without p63 or CK20 expression, whereas p63 and CK20 are often expressed in high‐grade urothelial carcinoma, with either a negative or positive P16 [6].

Despite its high malignancy, neuroendocrine carcinoma of the urinary bladder is often neglected due to its rarity. Previous studies [7, 8, 9] have demonstrated significant variation in prognosis for bladder NC. However, these studies relied solely on retrospective review and analysis of prognostic factors without adequately addressing confounding factors. Therefore, this study aimed to comprehensively compare NC with UC, the most common pathological subtype of bladder cancer. We analyzed the prognostic values of clinicopathological characteristics and survival outcomes in NC of the urinary bladder using the National Surveillance, Epidemiology, and End Results (SEER) database (2004–2015). Furthermore, to the best of our knowledge, this is the first study to assess prognosis differences in NC using propensity score matching (PSM) methods. PSM is widely acknowledged as a valuable tool in observational studies for minimizing confounding variables.

## 2 Materials and Methods

### 2.1 Patients

**FIGURE 1:**
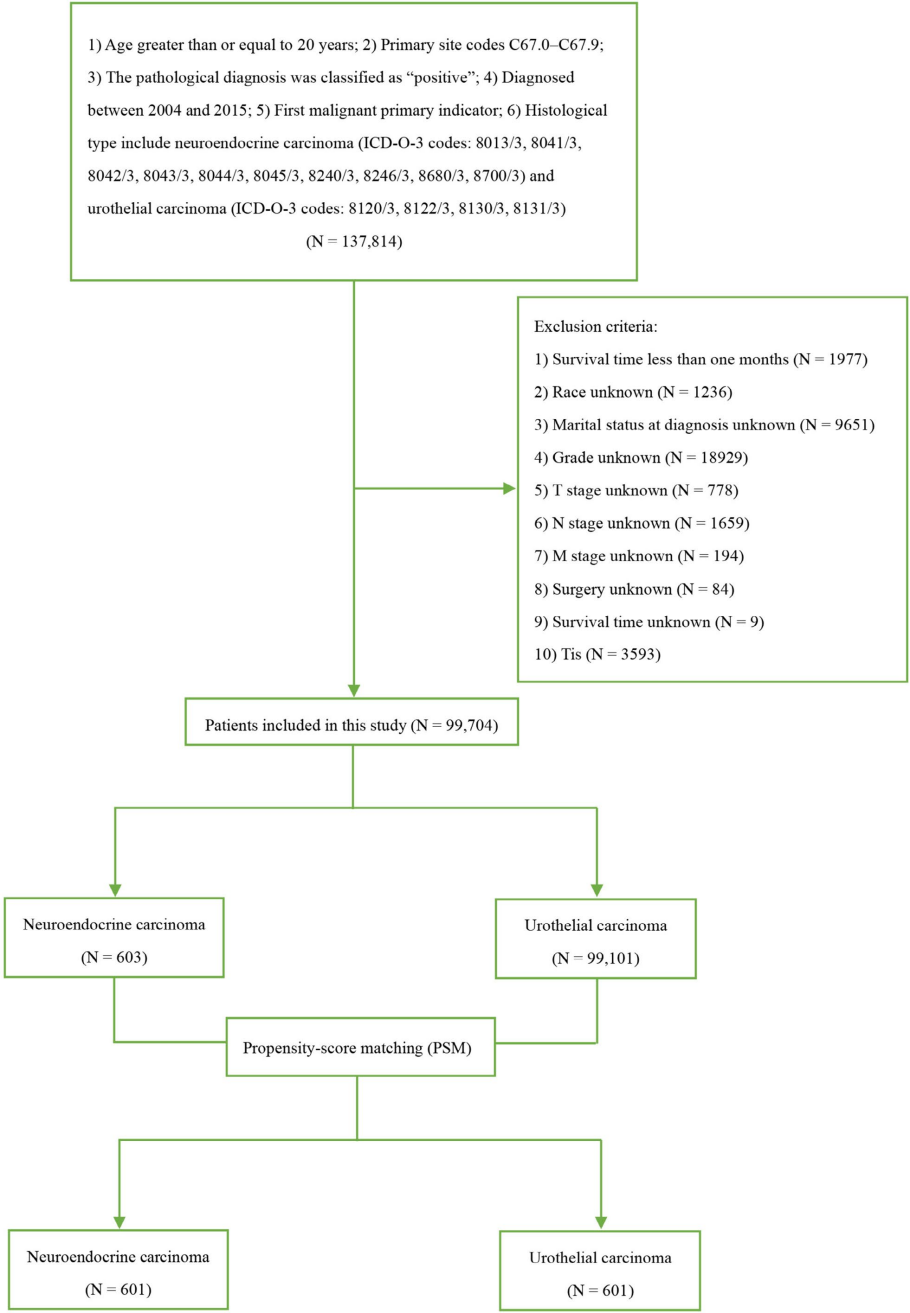
Patient selection flowchart.

Data for this study was obtained from the SEER*Stat 8.4.1.2 database, released on May 30, 2023. To be included, patients had to be at least 20 years old at diagnosis, have a primary tumor originating in the urinary bladder (site codes C67.0‐C67.9), possess a confirmed positive pathological diagnosis, receive a first‐time diagnosis of malignant cancer between 2004 and 2015, and have a documented histological type of neuroendocrine carcinoma (ICD‐O‐3 codes: 8013/3, 8041/3, 8042/3, 8043/3, 8044/3, 8045/3, 8240/3, 8246/3, 8680/3, 8700/3) and urothelial carcinoma (ICD‐O‐3 codes: 8120/3, 8122/3, 8130/3, 8131/3). Patients were excluded from the study if they met any of the following criteria: (1) survival time less than 1 month (*n* = 1977), (2) unknown race (*n* = 1236), (3) unknown marital status at diagnosis (*n* = 9651), (4) unknown grade/cT stage/cN stage/cM stage (*n* = 21,560), (5) unknown surgery status (*n* = 84), (6) unknown survival time (*n* = 9), or (7) diagnosis of bladder cancer in situ (Tis) (*n* = 3593) (Figure 1). The variables such as “surgery,” “chemotherapy,” and “radiation” could happen at any time during the entire treatment course of these patients. Given SEER's public nature, informed consent and institutional review board approval were not required. This study was conducted and reported in accordance with the Strengthening the Reporting of Cohort, Cross‐Sectional, and Case–Control Studies in Surgery (STROCSS) criteria [10].

### 2.2 Outcomes

The primary endpoints of the study were overall survival (OS) and cancer‐specific survival (CSS). OS was defined as the time elapsed from the initial diagnosis of bladder cancer to the date of the last follow‐up or death from any cause. CSS was defined as the time elapsed from the diagnosis of bladder cancer to death specifically attributable to the primary tumor or the date of the last follow‐up.

### 2.3 Propensity‐Score Matching (PSM)

Propensity score matching was employed to mitigate the influence of potential confounding variables arising from baseline clinicopathological differences between bladder cancer patients with NC and those with UC. This technique allows for a more accurate assessment of the independent impact of histological type on patient prognosis. PSM essentially balances these background differences by creating a standardized propensity score. This score incorporates the combined influence of multiple covariates, aiming to achieve comparable covariate distributions between the NC and UC groups. Similar to randomization in controlled trials, PSM helps balance confounding factors in non‐randomized studies, thereby reducing selection bias. The propensity score‐matched analysis in this study was based on the following variables: marital status at diagnosis, tumor grade, cTNM stage (cT stage, cN stage, and cM stage), surgery of the primary site, radiation therapy, and chemotherapy. A 1:1 greedy nearest neighbor matching algorithm was utilized within a caliper of 0.00001 for the propensity score. This strategy successfully matched 601 patients in each group (NC and UC).

### 2.4 Statistical Analysis

Categorical variables were analyzed using the Chi‐square test and are presented as percentages and frequencies. Univariate and multivariate Cox proportional hazards regression models were employed to identify risk factors for OS and CSS. Kaplan–Meier curves were generated to estimate OS and CSS, with differences between groups assessed using the log‐rank test. Statistical significance was defined as a two‐tailed *p*‐value less than 0.05. All statistical analyses were performed using SPSS version 25.0 and R 4.1.1.

## 3 Results

### 3.1 Baseline Characteristics Before and After PSM

A total of 99,704 patients were included in this study, with 603 (0.6%) diagnosed with NC and 99,101 (99.4%) diagnosed with UC. Within the NC group, the majority of patients were older adults (*n* = 502, 83.2%), male (*n* = 449, 74.5%), and white (*n* = 545, 90.4%). Additionally, 84.5% (*n* = 510) had muscle‐invasive bladder cancer at diagnosis. Notably, lymph node involvement (*n* = 129, 21.4%) and distant metastasis (*n* = 119, 19.7%) were less prevalent at initial diagnosis within the NC group. Regarding treatment, 89.3% (*n* = 538) of NC patients underwent either transurethral resection of the bladder (TURB) or radical cystectomy. Statistical analysis revealed significant differences between the NC and UC groups in marital status at diagnosis, tumor grade, cTNM stage (cT, cN, and cM stages), surgery of the primary site, radiation therapy, and chemotherapy (Table S1). Following propensity score matching, all baseline variables except race demonstrated no significant differences between the matched groups (Table 1).

**TABLE 1:** Clinicopathological characteristics of 1202 patients after PSM.

Variables	Total *N* = 1202	NC *N* = 601	UC *N* = 601	*p*
Age, year				
< 60 (%)	196 (16.3)	100 (16.6)	96 (16.0)	0.538
60–80 (%)	689 (57.3)	351 (58.4)	338 (56.2)	
≥ 80 (%)	317 (26.4)	150 (25.0)	167 (27.8)	
Sex				
Female (%)	331 (27.5)	154 (25.6)	177 (29.5)	0.138
Male (%)	871 (72.5)	447 (74.4)	424 (70.5)	
Race				
White (%)	1027 (85.4)	543 (90.3)	484 (80.5)	< 0.001
Black (%)	93 (7.7)	36 (6.0)	57 (9.5)	
Others (%)	82 (6.8)	22 (3.7)	60 (10.0)	
Marital status				
Married (%)	715 (59.5)	361 (60.1)	354 (58.9)	0.901
Single (%)	136 (11.3)	68 (11.3)	68 (11.3)	
SDW (%)	351 (29.2)	172 (28.6)	179 (29.8)	
T stage				
T0/Ta/T1 (%)	183 (15.2)	91 (15.1)	92 (15.2)	1.000
T2 (%)	660 (54.9)	330 (54.9)	330 (54.9)	
T3 (%)	225 (18.7)	113 (18.8)	112 (18.6)	
T4 (%)	134 (11.1)	67 (11.1)	67 (11.1)	
N stage				
N0 (%)	938 (78.0)	473 (78.7)	465 (77.4)	0.577
N+ (%)	264 (22.0)	128 (21.3)	136 (22.6)	
M stage				
M0 (%)	969 (80.6)	484 (80.5)	485 (80.7)	0.942
M1 (%)	233 (19.4)	117 (19.5)	116 (19.3)	
Surgery				
None (%)	25 (2.1)	10 (1.7)	15 (2.5)	0.758
TURB (%)	661 (55.0)	332 (55.2)	329 (54.7)	
RC (%)	412 (34.3)	205 (34.1)	207 (34.4)	
Others (%)	104 (8.7)	54 (9.0)	50 (8.3)	
Radiation				
None/unknown (%)	899 (74.8)	451 (75.0)	448 (74.5)	0.842
Yes (%)	303 (25.2)	150 (25.0)	153 (25.5)	
Chemotherapy				
None/unknown (%)	458 (38.1)	229 (38.1)	229 (38.1)	1.000
Yes (%)	744 (61.9)	372 (61.9)	372 (61.9)	
Survival status				
Alive (%)	206 (17.1)	94 (15.6)	112 (18.6)	0.168
Dead (%)	996 (82.9)	507 (84.4)	489 (81.4)	

Abbreviations: NC: neuroendocrine carcinoma; RC: partial cystectomy + simple/total/complete cystectomy + complete cystectomy with reconstruction + pelvic exenteration + cystectomy; SDW: separated + divorced + widowed; TURB: transurethral resection of the bladder; UC: urothelial carcinoma.

### 3.2 Survival Analysis Before and After PSM

**FIGURE 2:**
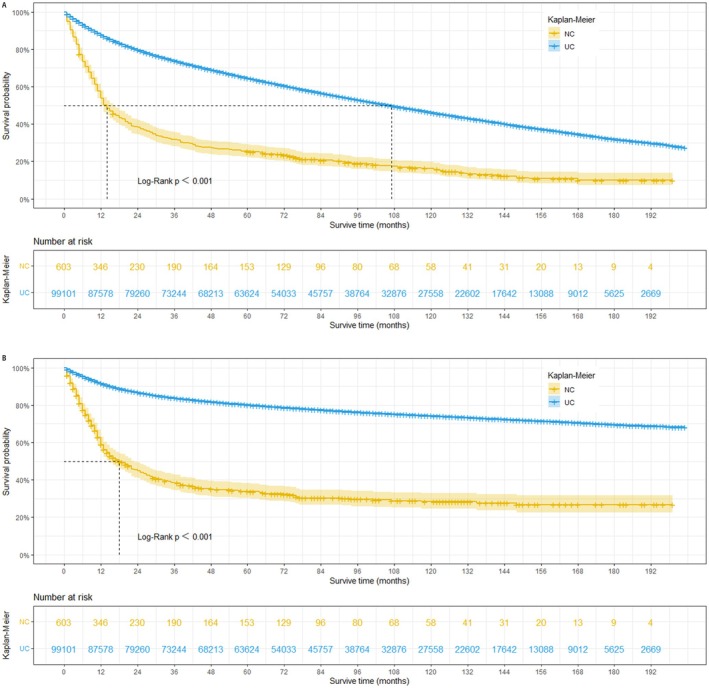
Overall survival (A) and cancer‐specific survival (B) of 99,704 unmatched patients in the NC group and UC group (NC: Neuroendocrine carcinoma; UC: Urothelial carcinoma).

**FIGURE 3:**
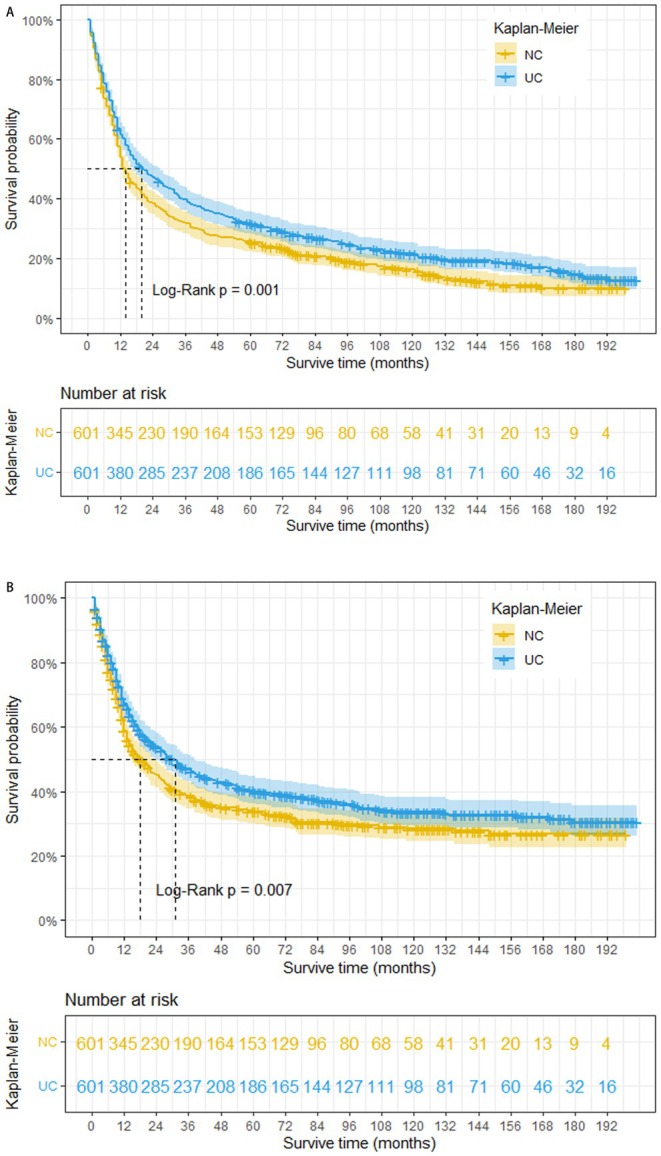
Overall survival (A) and cancer‐specific survival (B) of 1202 matched patients in the NC group and UC group (NC: Neuroendocrine carcinoma; UC: Urothelial carcinoma).

This study revealed that prior to PSM, patients with bladder NC exhibited significantly shorter OS and CSS compared to those with UC. The estimated median survival time for OS was 14 months in the NC group, compared to 107 months in the UC group (log‐rank *p* < 0.001). Similarly, the estimated median survival time for CSS was 18 months in the NC group, whereas it exceeded 200 months in the UC group (*Figure* *2* *A and* B). To mitigate potential bias arising from baseline differences, propensity score matching was performed at a 1:1 ratio. Following PSM, a total of 1202 patients were included in the matched groups. Our analysis demonstrated that even after adjusting for baseline characteristics, bladder NC remained significantly associated with worse OS and CSS. The median survival time for OS was 14 months in the NC group compared to 20 months in the UC group (log‐rank *p =* 0.001). Similarly, the median survival time for CSS was 14 months in the NC group compared to 31 months in the UC group (log‐rank *p* = 0.007) (*Figure* *3* *A and* B).

**FIGURE 4:**
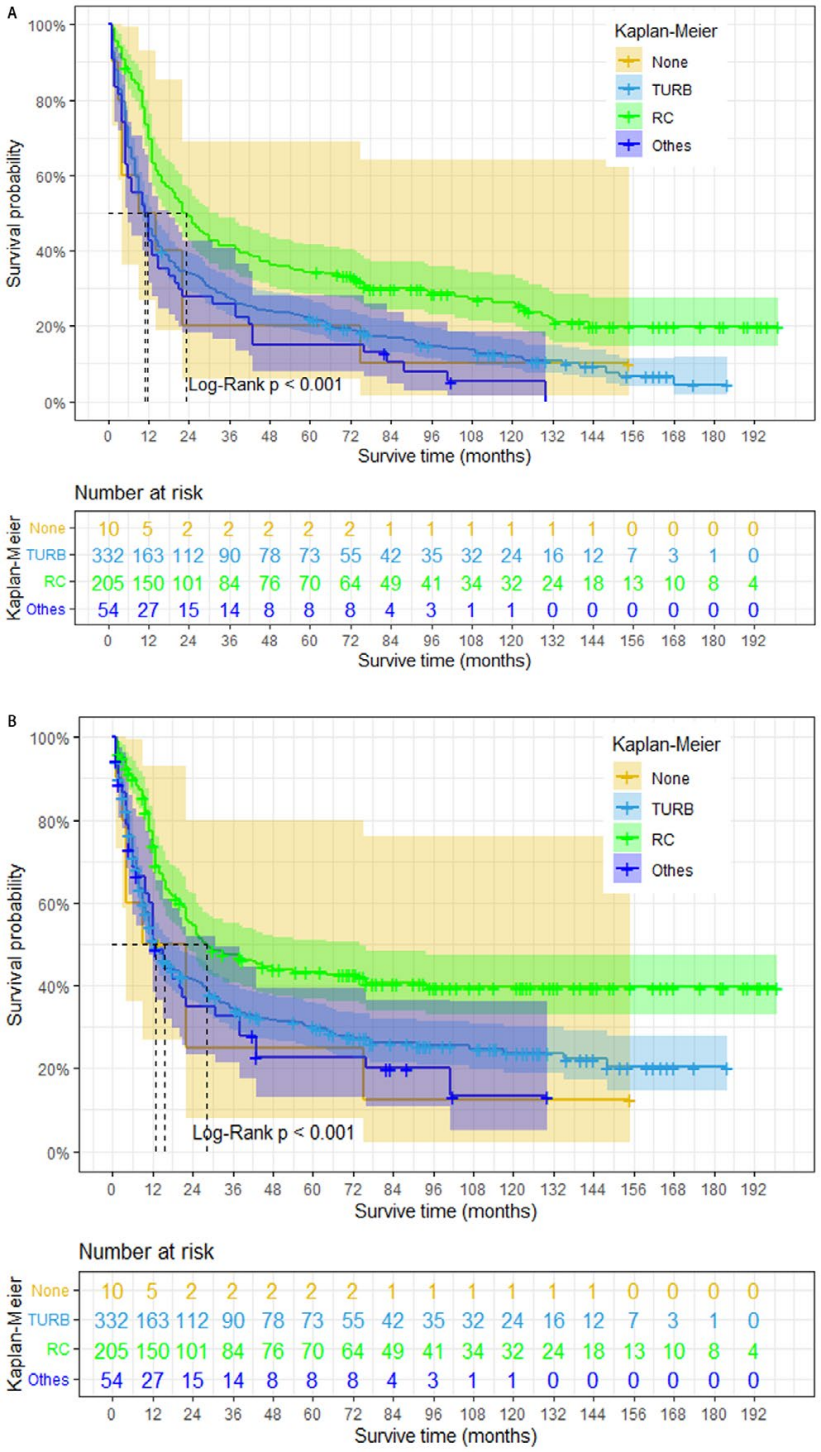
Overall survival (A) and cancer‐specific survival (B) in 601 NC patients (TURB: Transurethral resection of the bladder; RC: Partial cystectomy + simple/total/complete cystectomy + complete cystectomy with reconstruction + pelvic exenteration + cystectomy).

**FIGURE 5:**
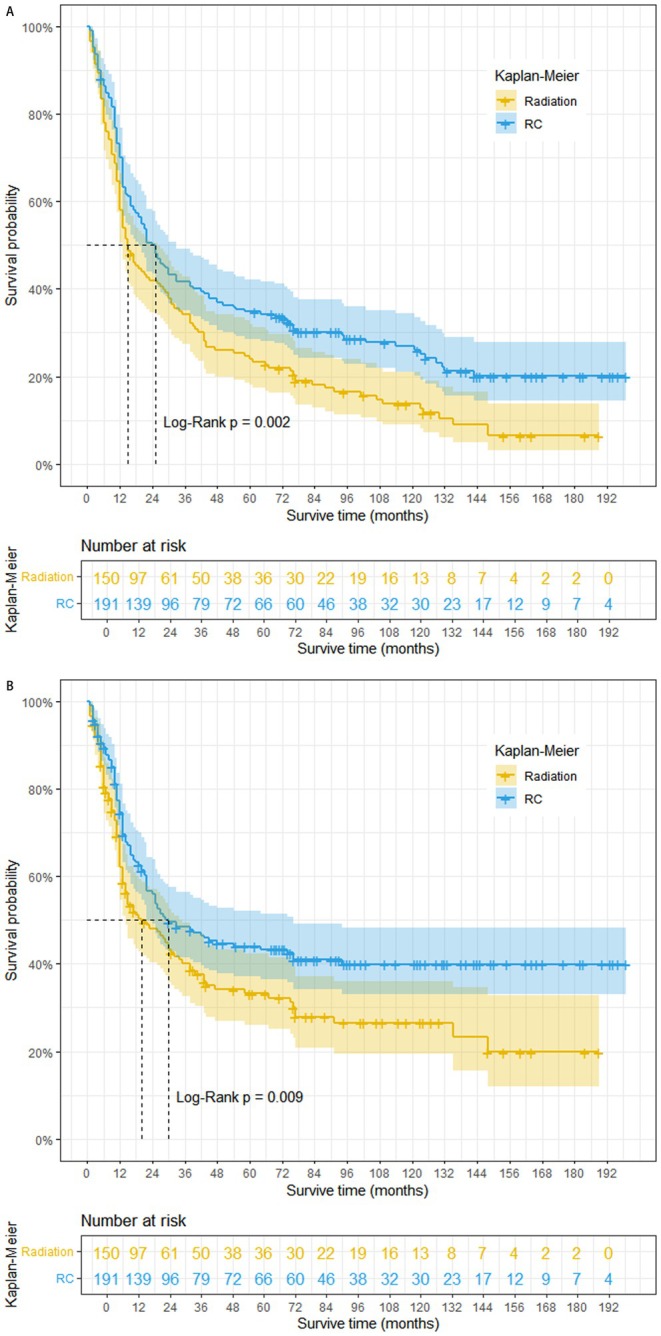
Overall survival (A) and cancer‐specific survival (B) of NC patients (RC: Partial cystectomy + simple/total/complete cystectomy + complete cystectomy with reconstruction + pelvic exenteration + cystectomy).

To further investigate the impact of surgery on NC prognosis, we generated Kaplan–Meier survival curves following PSM (Figure 4). Our analysis revealed that NC patients who received radical cystectomy have a better prognosis in both OS and CSS (log‐rank *p* < 0.001). However, patients who underwent radical cystectomy achieved demonstrably better survival outcomes compared to those who did not undergo surgery (median OS: 23 months vs. 9 months; median CSS: 28 months vs. 9 months). The median OS for patients who underwent TURB or other surgeries was 11 months for both, with a median CSS of 13 months for both groups. Besides, we also found NC patients who only received radical cystectomy have a better prognosis in both OS (log‐rank *p* = 0.002) and CSS (log‐rank *p* = 0.009) compared with those who only received radiotherapy (Figure 5).

### 3.3 Independent Prognostic Factors for OS and CSS Before PSM

Cox proportional hazards regression models were employed to evaluate prognostic factors for OS and CSS. Initially, all eleven baseline variables were incorporated into the univariate analysis (Tables S2 and S3). Ten of these variables demonstrated statistically significant associations (*p* < 0.05) with both OS and CSS: age at diagnosis, race, marital status, histological type, cTNM stage (cT, cN, and cM stages), surgery of the primary site, radiation therapy, and chemotherapy. Additionally, gender emerged as a significant risk factor for CSS in the univariate analysis. Subsequently, variables with statistically significant *p*‐values in the univariate analysis were included in the multivariate analysis (Tables S2 and S3). The multivariate Cox regression analysis revealed that ten variables remained significantly associated with both OS and CSS: age at diagnosis, race, marital status, histological type, cTNM stage, surgery of the primary site, radiation therapy, and chemotherapy.

### 3.4 Independent Prognostic Factors for OS and CSS After PSM

PSM yielded a different prognostic factor profile. Univariate analysis identified age at diagnosis (OS: 60–80 year vs. < 60 year, HR = 1.373, 95% CI = 1.139–1.655, *p* < 0.001; ≥ 80 year vs. < 60 year, HR = 2.315, 95% CI = 1.889–2.836, *p* < 0.001. CSS: ≥ 80 year vs. < 60 year, HR = 1.798, 95% CI = 1.435–2.253, *p* < 0.001), race (OS: others vs. white, HR = 0.704, 95% CI = 0.543–0.913, *p* = 0.008. CSS: others vs. white, HR = 0.668, 95% CI = 0.489–0.913, *p* = 0.011), marital status (OS: single vs. married, HR = 1.226, 95% CI = 1.003–1.499, *p* = 0.047; SDW vs. married, HR = 1.383, 95% CI = 1.204–1.588, *p* < 0.001. CSS: single vs. married, HR = 1.298, 95% CI = 1.036–1.627, *p* = 0.023; SDW vs. married, HR = 1.323, 95% CI = 1.126–1.555, *p* = 0.001), histological type (OS: NC vs. UC, HR = 1.222, 95% CI = 1.097–1.385, *p* = 0.002. CSS: NC vs. UC, HR = 1.216, 95% CI = 1.053–1.404, *p* = 0.008), cT stage (OS: T2 vs. T0/Ta/T1, HR = 1.178, 95% CI = 0.981–1.415, *p* = 0.079; T4 vs. T0/Ta/T1, HR = 1.851, 95% CI = 1.435–2.358, *p* = 0.001. CSS: T2 vs. T0/Ta/T1, HR = 1.388, 95% CI = 1.066–1.678, *p* = 0.012; T3 vs. T0/Ta/T1, HR = 1.488, 95% CI = 1.148–1.930, *p* = 0.003; T4 vs. T0/Ta/T1, HR = 2.383, 95% CI = 1.796–3.161, *p* < 0.001), cN stage (OS: NX vs. N0, HR = 1.698, 95% CI = 1.468–1.963, *p* < 0.001. CSS: NX vs. N0, HR = 2.009, 95% CI = 1.712–2.358, *p* < 0.001), cM stage (OS: M1 vs. M0, HR = 2.950, 95% CI = 2.528–3.444, *p* < 0.001. CSS: M1 vs. M0, HR = 3.470, 95% CI = 2.938–4.100, *p* < 0.001), surgery of the primary site (OS: TURBT vs. none, HR = 0.544, 95% CI = 0.362–0.819, *p* = 0.004; RC vs. none, HR = 0.312, 95% CI = 0.206–0.473, *p* < 0.001; others vs. none, HR = 0.581, 95% CI = 0.371–0.910, *p* = 0.018. CSS: TURBT vs. none, HR = 0.569, 95% CI = 0.355–0.912, *p* = 0.019; RC vs. none, HR = 0.322, 95% CI = 0.199–0.522, *p* < 0.001; others vs. none, HR = 0.564, 95% CI = 0.335–0.948, *p* = 0.031), and chemotherapy (OS: yes vs. none/unknown, HR = 0.753, 95% CI = 0.647–0.835, *p* < 0.001; CSS: yes vs. none/unknown, HR = 0.778, 95% CI = 0.671–0.902, *p* = 0.001) as significant prognostic factors for both OS and CSS (Tables 2 and 3). Notably, radiation therapy emerged as a prognostic factor solely for OS (yes vs. none/unknown, HR = 1.216, 95% CI = 1.057–1.399, *p* = 0.006). Multivariate Cox regression analysis in the PSM‐matched cohort of 1202 bladder cancer patients revealed seven independent prognostic factors for both OS and CSS: age at diagnosis (OS: 60–80 year vs. < 60 year, HR = 1.379, 95% CI = 1.141–1.667, *p* = 0.001; ≥ 80 year vs. < 60 year, HR = 2.007, 95% CI = 1.619–2.487, *p* < 0.001. CSS: ≥ 80 year vs. < 60 year, HR = 1.596, 95% CI = 1.257–2.028, *p* < 0.001), race (OS: others vs. white, HR = 0.674, 95% CI = 0.518–0.878, *p* = 0.003. CSS: others vs. white, HR = 0.622, 95% CI = 0.453–0.853, *p* = 0.003), cT stage (OS: T2 vs. T0/Ta/T1, HR = 1.340, 95% CI = 1.110–1.618, *p* = 0.002; T3 vs. T0/Ta/T1, HR = 1.745, 95% CI = 1.367–2.227, *p* < 0.001; T4 vs. T0/Ta/T1, HR = 1.955, 95% CI = 1511–2.529, *p* < 0.001. CSS: T2 vs. T0/Ta/T1, HR = 1.506, 95% CI = 1.194–1.899, *p* = 0.001; T3 vs. T0/Ta/T1, HR = 2.063, 95% CI = 1.545–2.755, *p* < 0.001; T4 vs. T0/Ta/T1, HR = 2.299, 95% CI = 1.707–3.095, *p* < 0.001), cN stage (OS: NX vs. N0, HR = 1.819, 95% CI = 1.542–2.144, *p* < 0.001. CSS: NX vs. N0, HR = 2.028, 95% CI = 1.693–2.430, *p* < 0.001), cM stage (OS: M1 vs. M0, HR = 2.453, 95% CI = 2.061–2.918, *p* < 0.001. CSS: M1 vs. M0, HR = 2.847, 95% CI = 2.360–3.435, *p* < 0.001), histological type (OS: NC vs. UC, HR = 1.253, 95% CI = 1.104–1.42, *p* = 0.001. CSS: NC vs. UC, HR = 1.239, 95% CI = 1.070–1.434, *p* = 0.004), and chemotherapy (OS: yes vs. none/unknown, HR = 0.648, 95% CI = 0.562–0.748, *p* < 0.001; CSS: yes vs. none/unknown, HR = 0.630, 95% CI = 0.537–0.741, *p* < 0.001) (Tables 2 and 3). Additionally, surgery of the primary site (RC vs. none, HR = 0.586, 95% CI = 0.371–0.925, *p* = 0.022) was identified as a significant prognostic factor for OS.

**TABLE 2:** Univariate and multivariate overall survival analyses of 1202 patients.

Variable	Univariate			Multivariate		
	HR	95% CI	*p*	HR	95% CI	*p*
Age, year						
< 60	Reference			Reference		
60–80	1.373	1.139–1.655	0.001	1.379	1.141–1.667	0.001
≥ 80	2.315	1.889–2.836	< 0.001	2.007	1.619–2.487	< 0.001
Sex						
Female	Reference					
Male	1.024	0.890–1.177	0.742			
Race						
White	Reference			Reference		
Black	1.102	0.880–1.378	0.398	1.095	0.867–1.382	0.448
Others	0.704	0.543–0.913	0.008	0.674	0.518–0.878	0.003
Marital status						
Married	Reference			Reference		
Single	1.226	1.003–1.499	0.047	1.133	0.918–1.398	0246
SDW	1.383	1.204–1.588	< 0.001	1.126	0.974–1.303	0.109
T stage						
T0/Ta/T1	Reference			Reference		
T2	1.178	0.981–1.415	0.079	1.340	1.110–1.618	0.002
T3	1.207	0.972–1.499	0.088	1.745	1.367–2.227	< 0.001
T4	1.851	1.453–2.358	0.001	1.955	1.511–2.529	< 0.001
N stage						
N0	Reference			Reference		
N+	1.698	1.468–1.963	< 0.001	1.819	1.542–2.144	< 0.001
M Stage						
M0	Reference			Reference		
M1	2.950	2.528–3.444	< 0.001	2.453	2.061–2.918	< 0.001
Surgery						
None	Reference			Reference		
TURB	0.544	0.362–0.819	0.004	1.083	0.702–1.671	0.719
RC	0.312	0.206–0.473	< 0.001	0.586	0.371–0.925	0.022
Others	0.581	0.371–0.910	0.018	1.322	0.822–2.127	0.249
Radiation						
None/unknown	Reference			Reference		
Yes	1.216	1.057–1.399	0.006	0.977	0.832–1.147	0.778
Chemotherapy						
None/unknown	Reference			Reference		
Yes	0.735	0.647–0.835	< 0.001	0.648	0.562–0.748	< 0.001
Histological type						
UC	Reference			Reference		
NC	1.222	1.079–1.385	0.002	1.253	1.104–1.423	0.001

Abbreviations: CI: confidence interval; CSS: cancer‐specific survival; HR: hazard ratio; NC: neuroendocrine carcinoma; OS: overall survival; RC: partial cystectomy + simple/total/complete cystectomy + complete cystectomy with reconstruction + pelvic exenteration + cystectomy; SDW: separated + divorced + widowed; TURB: transurethral resection of the bladder; UC: urothelial carcinoma.

**TABLE 3:** Univariate and multivariate cancer specific survival analyses of 1202 patients.

Variable	Univariate			Multivariate		
	HR	95% CI	*p*	HR	95% CI	*p*
Age, year						
< 60	Reference			Reference		
60–80	1.133	0.923–1.391	0.231	1.153	0.936–1.420	0.179
≥ 80	1.798	1.435–2.253	< 0.001	1.596	1.257–2.028	< 0.001
Sex						
Female	Reference					
Male	0.998	0.849–1.172	0.980			
Race						
White	Reference			Reference		
Black	1.176	0.916–1.511	0.203	1.148	0.884–1.489	0.300
Others	0.668	0.489–0.913	0.011	0.622	0.453–0.853	0.003
Marital status						
Married	Reference			Reference		
Single	1.298	1.036–1.627	0.023	1.108	0.875–1.404	0.395
SDW	1.323	1.126–1.555	0.001	1.078	0.910–1.276—	0.384
T stage						
T0/Ta/T1	Reference			Reference		
T2	1.338	1.066–1.678	0.012	1.506	1.194–1.899	0.001
T3	1.488	1.148–1.930	0.003	2.063	1.545–2.755	< 0.001
T4	2.383	1.796–3.161	< 0.001	2.299	1.707–3.095	< 0.001
N stage						
N0	Reference			Reference		
N+	2.009	1.712–2.358	< 0.001	2.028	1.693–2.430	< 0.001
M stage						
M0	Reference			Reference		
M1	3.470	2.938–4.100	< 0.001	2.847	2.360–3.435	< 0.001
Surgery						
None	Reference			Reference		
TURB	0.569	0.355–0.912	0.019	1.241	0.748–2.058	0.403
RC	0.322	0.199–0.522	< 0.001	0.650	0.384–1.100	0.108
Others	0.564	0.335–0.948	0.031	1.475	0.845–2.575	0.172
Radiation						
None/unknown	Reference					
Yes	1.161	0.988–1.365	0.070			
Chemotherapy						
None/unknown	Reference			Reference		
Yes	0.778	0.671–0.902	0.001	0.630	0.537–0.741	< 0.001
Histological type						
UC	Reference			Reference		
NC	1.216	1.053–1.404	0.008	1.239	1.070–1.434	0.004

Abbreviations: CI: confidence interval; CSS: cancer‐specific survival; HR: hazard ratio; NC: neuroendocrine carcinoma; OS: overall survival; RC: partial cystectomy + simple/total/complete cystectomy + complete cystectomy with reconstruction + pelvic exenteration + cystectomy; SDW: separated + divorced + widowed; TURB: transurethral resection of the bladder; UC: urothelial carcinoma.

## 4 Discussion

Bladder neuroendocrine carcinoma is a rare malignancy that presents significant challenges in establishing accurate prognoses. Additionally, the complex and variable treatment landscape for this disease hinders the prediction of both prognostic factors and treatment efficacy.

To our knowledge, this represents the first SEER analysis to utilize PSM to identify prognostic factors influencing OS and CSS specifically for bladder NC patients. The study yielded several noteworthy findings. Kaplan–Meier survival curves were generated to assess survival outcomes. Our model effectively identified independent risk factors for BCa patients, particularly those with NC. Notably, the analysis revealed a significantly worse prognosis for bladder NC compared to urothelial carcinoma. Based on the unadjusted survival curves (Figure 2A), nearly half of the NC patients did not survive beyond 14 months, while over 50% of urothelial carcinoma patients lived for 9 years or more. Furthermore, by comparing the survival curves in Figure 2, it can be inferred that most NC patient deaths were attributable to bladder cancer. The stratified analysis revealed that bladder NC patients who underwent radical cystectomy alone exhibited poorer OS and CSS compared to urothelial carcinoma patients. To account for potential confounding variables and derive more reliable conclusions, we subsequently performed propensity score matching. Our study revealed significantly poorer outcomes for bladder NC patients compared to UC patients. Based on the unadjusted survival curves (Figures 2 and 3), the median OS and CSS for bladder NC patients were 14 months and 18 months, respectively, while those for UC patients exceeded 9 years before PSM. This disparity could be attributed to the typically advanced stage (II–IV) at diagnosis for bladder NC patients, often accompanied by lymph node and distant metastases. To mitigate bias, we employed propensity score matching analysis. While PSM resulted in a substantial reduction in the median OS and CSS of bladder UC patients, their survival rates remained superior to those of bladder NC patients (OS: 20 months vs. 14 months, log‐rank *p* = 0.001; CSS: 31 months vs. 18 months, log‐rank *p* = 0.007). Before PSM, bladder NC patients who underwent no surgery, TURB, radical cystectomy, or other surgical procedures exhibited poorer OS and CSS compared to bladder UC patients who received the same treatments. Following PSM, bladder NC patients who underwent other surgical modalities displayed a worse OS than bladder UC patients.

Given the rarity of bladder NC, most recent studies have been limited to case reports [11, 12, 13]. Our findings based on the SEER database analysis are consistent with prior research utilizing SEER data and case series [7, 8, 14, 15]. One cohort study by Li et al. [7] identified TNM stage, marital status, age, chemotherapy, and surgery as independent prognostic factors. Previous studies have highlighted the significant heterogeneity in prognosis observed for bladder NC [7, 8, 9]. A small sample study by Zhou et al. [9] reported a more favorable prognosis for bladder NC, with 3‐year and 5‐year OS of 74.36% and 69.23%, respectively. In contrast, a study by Sroussi et al. [8] demonstrated a decline in OS with advancing stage for bladder NC patients. Their study included 236 patients diagnosed between 1997 and 2017, with 173 classified as early‐stage NC and 63 classified as advanced‐stage NC. The median OS for patients in stages I, II, IIIa, and IIIb was 36 months (95% CI, 29–43 months), 36 months (95% CI, 29–43 months), 26 months (95% CI, 18‐NR), and 16 months (95% CI, 12–21 months), respectively. Compared to the studies by Zhou et al. and Sroussi et al., our investigation boasts a substantially larger sample size (603 vs. 236 vs. 39). Additionally, our study population exhibited a higher rate of radiation therapy (151/603 vs. 30/236) compared to Sroussi et al. However, a lower proportion of our patients underwent radical cystectomy compared to Sroussi's study (116/236 vs. 206/603). The higher prevalence of TNM stage, muscle invasion, lymph node metastasis, and distant metastasis observed in bladder NC patients likely contributes to their poorer prognosis. To minimize the influence of confounding factors, our analysis employed propensity score‐matched data. We identified age at diagnosis, race, cTNM stage, chemotherapy, and histological type as significant prognostic factors influencing both OS and CSS, while surgery was found to impact OS only. Consistent with previous research, advanced age was associated with an increased risk of poor prognosis in bladder NC patients [16, 17]. The confirmation of higher rates of muscle‐invasive disease, lymph node metastasis, and distant metastasis in the TNM stage further supports the association with a poor prognosis for NC patients. During the follow‐up period, 509 (84.4%) patients succumbed to the disease, with a median OS and CSS of only 14 months and 18 months, respectively. Another large study by Li et al. [13] reported similar findings, with a median OS of 13 months.

Additionally, advanced stages suggest a more aggressive tumor with a higher likelihood of vascular invasion and metastasis. The analysis revealed that radical cystectomy (HR = 0.586, 95% CI = 0.371–0.925, *p* = 0.022) and chemotherapy (HR = 0.648, 95% CI = 0.562–0.748, *p* < 0.001) emerged as independent protective factors for OS after PSM. While radical cystectomy (HR = 0.650, 95% CI = 0.384–1.100, *p* = 0.0108) did not retain significance for CSS after PSM, chemotherapy (HR = 0.630, 95% CI = 0.537–0.741, *p* < 0.001) remained an independent protective factor. While surgery alone may not be sufficient [18, 19], it plays a crucial role in patient management. Platinum‐based neoadjuvant chemotherapy has been shown to improve outcomes [19], and data from the National Cancer Database suggests that radical cystectomy combined with chemotherapy and radiation offers superior survival compared to single‐modality therapy [18]. Our PSM analysis included 601 patients, of which 332 (55.2%) received TURB and only 205 (34.1%) underwent RC. This distribution could be attributed to the early stage of disease diagnosis (over 70% were T2 or lower) and potential patient preference for preserving quality of life by avoiding radical cystectomy, especially for those with a life expectancy exceeding 5 years. Consistent with prior retrospective studies on bladder cancer patients with NC, our analysis revealed a higher OS rate for those who received chemotherapy compared to those who did not [8, 20, 21]. Treatment regimens often mirrored those used for lung cancers with similar pathologies, with etoposide and platinum being commonly employed as neoadjuvant or adjuvant therapies [19, 22]. Neoadjuvant chemotherapy was used in the treatment of NC of the urinary tract in 2004, and they found 78% of patients have a better prognostic [23]. Then a phase 2 clinical trial [24] and a retrospective study [25] also confirm this finding. Recently, a phase 2 trial [26] using doxorubicin‐ifosfamide and cis‐platinum plus etoposide found that neoadjuvant chemotherapy can down‐stage the tumor (49.6% vs. 14.5%, *p* < 0.001) compared with initial surgery for NC of the urinary tract. Besides, they also found neoadjuvant chemotherapy was associated with improved survival when compared with urothelial therapy (145.4 months vs. 42.5 months, HR = 0.49, 95% CI = 0.25–0.94). A retrospective study with 216 patients with NC of the urothelial tract enrolled found clinical staging, pathologic staging, and morphology were strongly associated with recurrence [27].

In contrast to the majority of bladder cancers, our study population predominantly consisted of patients with muscle‐invasive bladder NC [28]. Despite exhibiting highly malignant, dedifferentiated, and aggressive characteristics, the underlying mechanisms driving NC development remain unclear. Research identified three potentially targetable pathways in genitourinary neuroendocrine tumors: the p53/Rb pathway, receptor tyrosine kinase signaling, and epigenetic regulators [29, 30]. These findings suggest promising avenues for novel therapeutic strategies. Furthermore, bladder NC displayed a high mutation rate (12.91 mutations per million base pairs), comparable to that observed in small‐cell lung cancer and muscle‐invasive bladder urothelial carcinoma [29, 31]. This shared characteristic suggests that cancer immunotherapies may hold significant efficacy in treating malignancies with high mutational burdens. The study period witnessed a growing trend towards multimodal treatment regimens for bladder NC patients. The approval of anti‐PD‐L1 and anti‐PD‐1 antibodies has provided new treatment options for patients ineligible for cisplatin‐based chemotherapy or those who have experienced treatment failure [32, 33]. Clinical trials are also exploring the use of dual checkpoint inhibitors for non‐urothelial bladder cancer [34, 35, 36]. Immune checkpoint inhibition is emerging as a potential therapeutic approach for non‐urothelial bladder cancer, mirroring its established role as a first‐line or second‐line treatment for urothelial carcinomas [37].

Our study offers several advantages compared to previous research. First, we leveraged a large sample size, enrolling 603 patients with NC and 99,101 patients with UC of the urinary bladder diagnosed between 2004 and 2015. This robust sample enabled us to conduct more precise and multifaceted analyses. Second, we implemented PSM for the first time in this context to mitigate the influence of confounding factors, thereby enhancing the data's reliability. Furthermore, we reviewed the clinicopathological characteristics of SRCC and its associated survival outcomes, informed by the latest available data. However, our study also has limitations. The retrospective nature of our analysis, utilizing the SEER database, inherently introduces some bias, even after employing PSM. To definitively address this limitation, a prospective controlled trial is warranted. Additionally, the SEER database lacks detailed information regarding specific radiotherapy and chemotherapy regimens, as well as newer therapies like checkpoint‐inhibitor drugs. The variable efficacy of different treatment options could potentially confound our findings.

## 5 Conclusion

Our analysis identified some prognostic factors influencing both OS and CSS in bladder NC patients. Compared to patients who did not undergo surgery or received other surgical procedures, those who underwent radical cystectomy for NC exhibited improved clinical outcomes. Therefore, radical cystectomy may represent the most favorable therapeutic option for improving the prognosis of NC patients.

## Author Contributions

**Liang Liu:** conceptualization (lead), data curation (lead), formal analysis (lead), funding acquisition (lead), investigation (lead), methodology (lead), project administration (lead), resources (lead), software (lead), supervision (lead), validation (lead), visualization (lead), writing – original draft (lead), writing – review and editing (lead). **Pan‐Ying Zhang:** data curation (equal), methodology (equal), software (equal), validation (equal), writing – review and editing (equal). **Yu Xiao:** data curation (equal), formal analysis (equal), software (equal), writing – original draft (equal), writing – review and editing (equal). **Qiang Wang:** data curation (equal), investigation (equal), methodology (equal), software (equal), writing – original draft (equal). **Ji Zheng:** data curation (equal), methodology (equal), software (equal), supervision (equal), writing – original draft (equal), writing – review and editing (equal).

## Disclosure

The authors verify that all information and materials in the manuscript are original.

## Ethics Statement

This is an observational study, and no ethical approval is required. All procedures performed in studies involving human participants were in accordance with the ethical standards of the institutional and national research committee and with the 1964 Helsinki Declaration and its later amendments or comparable ethical standards. The SEER Program collects data from population‐based cancer registries with anonymous information.

## Consent

The data was sourced from the SEER database, and appropriate measures have been taken to protect patient confidentiality, so no informed consent is required.

## Conflicts of Interest

The authors declare no conflicts of interest.

## Supporting information


Data S1.

## Data Availability

The datasets analyzed during the current study are available in the Surveillance, Epidemiology, and End Results (SEER) database repository [www.seer.cancer.gov].
