# Supplementary material for: Prognostic Factors Influencing Postoperative Survival in Patients With Neuroendocrine Carcinoma of the Bladder: A Population‐Based Study
Source: Cancer Med. 2025 Mar 10;14(5):e70758. doi: 10.1002/cam4.70758 (PMC11891926; doi:10.1002/cam4.70758)
Supplement: Supplementary file 1 — Data S1. [file CAM4-14-e70758-s001.docx]

**Supplementary Table 1** Clinicopathological characteristics of 99,704 patients before PSM

| **Variables** | **Total**  **N = 99,704** | **NC**  **N = 603** | **UC**  **N = 99,101** | ***P* value** |
| --- | --- | --- | --- | --- |
| Age, year |  |  |  | 0.169 |
| ＜60 (%) | 19,662 (19.7) | 101 (16.7) | 19,561 (19.7) |  |
| 60-80 (%) | 56,630 (56.8) | 351 (58.2) | 56,279 (56.8) |  |
| ≥80 (%) | 23,412 (23.5) | 151 (25.0) | 23,261 (23.5) |  |
| Sex |  |  |  | 0.254 |
| Female (%) | 23,505 (23.6) | 154 (25.5) | 23,351 (23.6) |  |
| Male (%) | 76,199 (76.4) | 449 (74.5) | 75,750 (76.4) |  |
| Race |  |  |  | 0.231 |
| White (%) | 89,978 (90.2) | 545 (90.4) | 89,433 (90.2) |  |
| Black (%) | 4,942 (5.0) | 36 (6.0) | 4,906 (5.0) |  |
| Others (%) | 4,784 (4.8) | 22 (3.6) | 4,762 (4.8) |  |
| Marital Status |  |  |  | 0.026 |
| Married (%) | 64,220 (64.4) | 362 (60.0) | 63,858 (64.4) |  |
| Single (%) | 11,523 (11.6) | 68 (11.3) | 11,455 (11.6) |  |
| SDW (%) | 23,961 (24.0) | 173 (28.7) | 23,788 (24.0) |  |
| T Stage |  |  |  | ＜0.001 |
| T0/Ta/T1 (%) | 76,370 (76.6) | 93 (15.4) | 76,277 (77.0) |  |
| T2 (%) | 15,133 (16.2) | 330 (54.7) | 14,803 (14.9) |  |
| T3 (%) | 4,620 (4.6) | 113 (18.7) | 4,507 (4.5) |  |
| T4 (%) | 3,581 (3.6) | 67 (11.1) | 3,514 (3.5) |  |
| N Stage |  |  |  | ＜0.001 |
| N0 (%) | 95,209 (95.5) | 474 (78.6) | 94,735 (95.6) |  |
| N+ (%) | 4,495 (4.5) | 129 (21.4) | 4,366 (4.4) |  |
| M Stage |  |  |  | ＜0.001 |
| M0 (%) | 96,931 (97.2) | 484 (80.3) | 96,447 (97.3) |  |
| M1 (%) | 2,773 (2.8) | 119 (19.7) | 2,654 (2.7) |  |
| Surgery |  |  |  | ＜0.001 |
| None (%) | 2,679 (2.7) | 10 (1.7) | 2,669 (2.7) |  |
| TURB (%) | 69,333 (69.5) | 332 (55.1) | 69,001 (69.6) |  |
| RC (%) | 12,309 (12.3) | 206 (34.2) | 12,103 (12.2) |  |
| Others (%) | 15,383 (15.4) | 55 (9.1) | 15,328 (15.5) |  |
| Radiation |  |  |  | ＜0.001 |
| None/Unknown (%) | 94,954 (95.2) | 452 (75.0) | 94,502 (95.4) |  |
| Yes (%) | 4,750 (4.8) | 151 (25.0) | 4,599 (4.6) |  |
| Chemotherapy |  |  |  | ＜0.001 |
| None/Unknown (%) | 77,300 (77.5) | 229 (38.0) | 77,071 (77.8) |  |
| Yes (%) | 22,404 (22.5) | 374 (62.0) | 22,030 (22.2) |  |
| Survival Status |  |  |  | ＜0.001 |
| Alive (%) | 43,898 (44.0) | 94 (15.6) | 43,804 (44.2) |  |
| Dead (%) | 55,806 (56.0) | 509 (84.4) | 55,297 (55.8) |  |

**Note:** NC: neuroendocrine carcinoma; UC: urothelial carcinoma; SDW: separated + divorced + widowed; TURB: transurethral resection of the bladder; RC: partial cystectomy + simple/total/complete cystectomy + complete cystectomy with reconstruction + pelvic exenteration + cystectomy.

**Supplementary Table 2** Univariate and multivariate overall survival analyses of 99,704 patients

| **Variable** | **Univariate** | | | **Multivariate** | | |
| --- | --- | --- | --- | --- | --- | --- |
|  | **HR** | **95% CI** | ***P* value** | **HR** | **95% CI** | ***P* value** |
| Age, year |  |  |  |  |  |  |
| ＜60 | Reference |  |  | Reference |  |  |
| 60-80 | 2.400 | 2.332-2.470 | ＜0.001 | 2.433 | 2.364-2.505 | ＜0.001 |
| ≥80 | 6.293 | 6.200-6.589 | ＜0.001 | 6.168 | 5.978-6.364 | ＜0.001 |
| Sex |  |  |  |  |  |  |
| Female | Reference |  |  |  |  |  |
| Male | 1.004 | 0.984-1.024 | 0.712 |  |  |  |
| Race |  |  |  |  |  |  |
| White | Reference |  |  | Reference |  |  |
| Black | 1.228 | 1.184-1.274 | ＜0.001 | 1.179 | 1.136-1.224 | ＜0.001 |
| Others | 0.878 | 0.842-0.915 | ＜0.001 | 0.841 | 0.807-0.877 | ＜0.001 |
| Marital Status |  |  |  |  |  |  |
| Married | Reference |  |  | Reference |  |  |
| Single | 1.088 | 1.058-1.118 | ＜0.001 | 1.246 | 1.212-1.281 | ＜0.001 |
| SDW | 1.702 | 1.670-1.734 | ＜0.001 | 1.283 | 1.259-1.308 | ＜0.001 |
| T Stage |  |  |  |  |  |  |
| T0/Ta/T1 | Reference |  |  | Reference |  |  |
| T2 | 2.645 | 2.590-2.702 | ＜0.001 | 2.655 | 2.587-2.726 | ＜0.001 |
| T3 | 2.914 | 2.816-3.015 | ＜0.001 | 3.419 | 3.269-3.576- | ＜0.001 |
| T4 | 4.679 | 4.511-4.853 | ＜0.001 | 4.298 | 4.115-4.488 | ＜0.001 |
| N Stage |  |  |  |  |  |  |
| N0 | Reference |  |  | Reference |  |  |
| N+ | 3.608 | 3.491-3.728 | ＜0.001 | 1.804 | 1.734-1.878 | ＜0.001 |
| M Stage |  |  |  |  |  |  |
| M0 | Reference |  |  | Reference |  |  |
| M1 | 7.520 | 7.225-7.826 | ＜0.001 | 3.256 | 3.113-3.406 | ＜0.001 |
| Surgery |  |  |  |  |  |  |
| None | Reference |  |  | Reference |  |  |
| TURB | 0.799 | 0.761-0.840 | ＜0.001 | 0.881 | 0.838-0.927 | ＜0.001 |
| RC | 1.177 | 1.116-1.242 | ＜0.001 | 0.569 | 0.537-0.603 | ＜0.001 |
| Others | 0.773 | 0.733-0.815 | ＜0.001 | 0.887 | 0.841-0.936 | ＜0.001 |
| Radiation |  |  |  |  |  |  |
| None/Unknown | Reference |  |  | Reference |  |  |
| Yes | 3.461 | 3.353-3.573 | ＜0.001 | 1.202 | 1.159-1.247 | ＜0.001 |
| Chemotherapy |  |  |  |  |  |  |
| None/Unknown | Reference |  |  | Reference |  |  |
| Yes | 1.247 | 1.222-1.272 | ＜0.001 | 0.875 | 0.856-0.895 | ＜0.001 |
| Histological Type |  |  |  |  |  |  |
| UC | Reference |  |  | Reference |  |  |
| NC | 3.046 | 2.791-3.324 | ＜0.001 | 1.395 | 1.277-1.524 | ＜0.001 |

**Note:** NC: neuroendocrine carcinoma; UC: urothelial carcinoma; SDW: separated + divorced + widowed; TURB: transurethral resection of the bladder; RC: partial cystectomy + simple/total/complete cystectomy + complete cystectomy with reconstruction + pelvic exenteration + cystectomy; OS: overall survival; CSS: cancer-specific survival; HR: hazard ratio; CI: confidence interval.

**Supplementary Table 3** Univariate and multivariate cancer specific survival analyses of 99,704 patients

| **Variable** | **Univariate** | | | **Multivariate** | | |
| --- | --- | --- | --- | --- | --- | --- |
|  | **HR** | **95% CI** | ***P* value** | **HR** | **95% CI** | ***P* value** |
| Age, year |  |  |  |  |  |  |
| ＜60 | Reference |  |  | Reference |  |  |
| 60-80 | 1.555 | 1.495-1.617 | ＜0.001 | 1.603 | 1.541-1.668 | ＜0.001 |
| ≥80 | 3.274 | 3.319-3.414 | ＜0.001 | 3.258 | 3.117-3.405 | ＜0.001 |
| Sex |  |  |  |  |  |  |
| Female | Reference |  |  | Reference |  |  |
| Male | 0.852 | 0.828-0.877 | ＜0.001 | 1.002 | 0.972-1.034 | 0.892 |
| Race |  |  |  |  |  |  |
| White | Reference |  |  | Reference |  |  |
| Black | 1.550 | 1.473-1.632 | ＜0.001 | 1.271 | 1.207-1.339 | ＜0.001 |
| Others | 0.984 | 0.925-1.046 | 0.595 | 0.911 | 0.857-0.969 | 0.003 |
| Marital Status |  |  |  |  |  |  |
| Married | Reference |  |  | Reference |  |  |
| Single | 1.281 | 1.231-1.334 | ＜0.001 | 1.281 | 1.229-1.335 | ＜0.001 |
| SDW | 1.693 | 1.645-1.743 | ＜0.001 | 1.270 | 1.231-1.310 | ＜0.001 |
| T Stage |  |  |  |  |  |  |
| T0/Ta/T1 | Reference |  |  | Reference |  |  |
| T2 | 5.772 | 5.602-5.947 | ＜0.001 | 5.545 | 5.349-5.748 | ＜0.001 |
| T3 | 7.013 | 6.721-7.318 | ＜0.001 | 7.692 | 7.270-8.138- | ＜0.001 |
| T4 | 11.525 | 11.030-12.042 | ＜0.001 | 9.433 | 8.939-9.953 | ＜0.001 |
| N Stage |  |  |  |  |  |  |
| N0 | Reference |  |  | Reference |  |  |
| N+ | 6.663 | 6.418-6.916 | ＜0.001 | 1.937 | 1.850-2.027 | ＜0.001 |
| M Stage |  |  |  |  |  |  |
| M0 | Reference |  |  | Reference |  |  |
| M1 | 12.856 | 12.305-13.432 | ＜0.001 | 3.494 | 3.324-3.672 | ＜0.001 |
| Surgery |  |  |  |  |  |  |
| None | Reference |  |  | Reference |  |  |
| TURB | 0.672 | 0.623-0.723 | ＜0.001 | 0.840 | 0.779-0.905 | ＜0.001 |
| RC | 1.666 | 1.542-1.800 | ＜0.001 | 0.531 | 0.489-0.577 | ＜0.001 |
| Others | 0.584 | 0.539-0.634 | ＜0.001 | 0.805 | 0.741-0.873 | ＜0.001 |
| Radiation |  |  |  |  |  |  |
| None/Unknown | Reference |  |  | Reference |  |  |
| Yes | 5.076 | 4.882-5.278 | ＜0.001 | 1.227 | 1.173-1.283 | ＜0.001 |
| Chemotherapy |  |  |  |  |  |  |
| None/Unknown | Reference |  |  | Reference |  |  |
| Yes | 1.846 | 1.795-1.897 | ＜0.001 | 0.884 | 0.856-0.912 | ＜0.001 |
| Histological Type |  |  |  |  |  |  |
| UC | Reference |  |  | Reference |  |  |
| NC | 4.847 | 4.383-5.361 | ＜0.001 | 1.465 | 1.323-1.622 | ＜0.001 |

**Note:** NC: neuroendocrine carcinoma; UC: urothelial carcinoma; SDW: separated + divorced + widowed; TURB: transurethral resection of the bladder; RC: partial cystectomy + simple/total/complete cystectomy + complete cystectomy with reconstruction + pelvic exenteration + cystectomy; OS: overall survival; CSS: cancer-specific survival; HR: hazard ratio; CI: confidence interval.
